# Tumour-reactive T cell subsets in the microenvironment of ovarian cancer

**DOI:** 10.1038/s41416-019-0384-y

**Published:** 2019-02-05

**Authors:** Marie Christine Wulff Westergaard, Rikke Andersen, Chloé Chong, Julie Westerlin Kjeldsen, Magnus Pedersen, Christina Friese, Thomas Hasselager, Henrik Lajer, George Coukos, Michal Bassani-Sternberg, Marco Donia, Inge Marie Svane

**Affiliations:** 1 Center for Cancer Immune Therapy, Department of Hematology, Herlev Hospital, University of Copenhagen, Copenhagen, Denmark; 2 Department of Oncology, Herlev Hospital, University of Copenhagen, Copenhagen, Denmark; 3 Ludwig Institute for Cancer Research, University of Lausanne, Lausanne, Switzerland; 4 Department of Oncology, University Hospital of Lausanne, Lausanne, Switzerland; 5 Department of Pathology, Herlev Hospital, University of Copenhagen, Copenhagen, Denmark; 6 Department of Gynaecology, Rigshospitalet, University of Copenhagen, Copenhagen, Denmark

**Keywords:** Tumour immunology, Ovarian cancer, Ovarian cancer, Tumour immunology

## Abstract

**Background:**

Solid malignancies are frequently infiltrated with T cells. The success of adoptive cell transfer (ACT) with expanded tumour-infiltrating lymphocytes (TILs) in melanoma warrants its testing in other cancer types. In this preclinical study, we investigated whether clinical-grade TILs could be manufactured from ovarian cancer (OC) tumour specimens.

**Methods:**

Thirty-four tumour specimens were obtained from 33 individual patients with OC. TILs were analysed for phenotype, antigen specificity and functionality.

**Results:**

Minimally expanded TILs (Young TILs) were successfully established from all patients. Young TILs contained a high frequency of CD3^+^ cells with a variable CD4/CD8 ratio. TILs could be expanded to clinical numbers. Importantly, recognition of autologous tumour cells was demonstrated in TILs in >50% of the patients. We confirmed with mass spectrometry the presentation of multiple tumour antigens, including peptides derived from the cancer-testis antigen GAGE, which could be recognised by antigen-specific TILs. Antigen-specific TILs could be isolated and further expanded in vitro.

**Conclusion:**

These findings support the hypothesis that patients with OC can benefit from ACT with TILs and led to the initiation of a pilot clinical trial at our institution .

**Trial Registration:**

clinicaltrials.gov: NCT02482090.

## Introduction

The density of intratumoural infiltration with T cells has been associated with improved survival in virtually all forms of solid tumours evaluated.^1–4^ In fact, the natural infiltrates of solid tumours may contain T cells targeting several types of tumour antigens, including those derived from tumour-specific mutated proteins.^5,6^ A technically complex approach of personalised cancer immunotherapy based on the isolation, in vitro expansion and reinfusion of T cells isolated from the microenvironment of an individual patient’s tumour metastasis, has demonstrated promising results. However, so far current protocols of T cell expansion and patient conditioning have only been tested in a few selected forms of metastatic cancer.^7^

The human immune system naturally generates adaptive immune responses against ovarian cancer (OC).^8–10^ OC has a moderately high mutational load on average,^11^ and immune recognition of cancer mutations have previously been shown.^8^ Overall, OC appears to be an promising target for strategies enhancing endogenous immune responses.^12^

OC is the most lethal gynaecologic malignancy. It is often diagnosed at an advanced stage (e.g. International Federation of Gynecology and Obstetrics (FIGO) stage III or IV), which combined with the lack of curative medical treatment options,^13^ contributes to a poor relative 5 year survival rate of 39% and 17% in stage III and IV, respectively, for the most common epithelial carcinomas, which comprise 85–90% of cases.^14^ So far immunotherapy with checkpoint inhibitors has shown limited efficacy in OC, with response rates ranging from 6–17%.^15–18^ These observations prompted us to explore whether a personalised immunotherapy approach based on the expansion of tumour-infiltrating lymphocytes (TILs) would be feasible in patients with OC. In this study, we isolate, expand and test the antitumour activity in vitro of TILs obtained from a cohort of unselected patients with OC. We show that TIL cultures can easily be generated in the laboratory and display antitumour activities in the majority of cases. Further, we demonstrate that tumour antigen-specific TILs can be isolated and expanded, resulting in increased antitumour response.

## Patients, materials and methods

### Patient material

We obtained 34 metastatic (intraoperative evaluation) tumour specimens from 33 individual patients with histologically verified OC. For the vast majority of the patients the cancer was in an advanced stage (Supplementary Table 1). All surgical resections were part of either a primary cytoreductive or an interval debulking procedure for OC at the Department of Gynaecology, Rigshospitalet, Copenhagen. From one patient, tumour specimens were received twice with 16 months interval (total *n* = 34 samples). Ascitic fluid was collected from 11 patients. The tumour specimens were transported to Herlev Hospital immediately after surgery and processed within 2 h. The scientific use of the patient material was approved by the National Committee on Health Research Ethics (reference number H-2-2014-055).

### Reagents for TILs and tumour cells

Complete medium (CM) used for culturing TILs consisted of RPMI-1640 with GlutaMAX, 25 mM HEPES pH 7.2 (Gibco, Massachusetts, USA), 100 U/ml penicillin (Gibco), 100 μg/ml streptomycin (Gibco) and Fungizone® (Bristol-Myers Squibb, Middlesex, United Kingdom) 1.25 μg/ml supplemented with 10% Human AB Serum (Sigma-Aldrich, Missouri, United States) and 6000 IU/ml of rhIL-2 (Proleukin, Novartis, Copenhagen, Denmark).

Rapid expansion medium (RM) used for the rapid expansion protocol (REP) consisted of AIM-V medium (Gibco) and Fungizone 1.25 μg/ml supplemented with 6000 IU/ml rhIL-2.

In addition, OKT3 (anti-CD3) antibody (MACs Miltenyi Biotech, Bergisch Gladbach, Germany), Pulmozyme (Roche, Basel, Switzerland), and allogeneic peripheral blood mononuclear cells (PBMCs) (or feeder cells) obtained from buffy coats from healthy donors are used in the REP.

R10 medium used for culturing tumour cells consisted of RPMI-1640 with GlutaMAX, 25 mM HEPES pH 7.2, 100 U/ml penicillin, 100 μg/ml streptomycin and foetal bovine serum (FBS) (Gibco). In addition, Solu-Cortef (hydrocortisone sodium succinate) (the local hospital pharmacy) 500 ng/ml was added to R10, the first month of establishing the tumour cell line (TCL).

Enzyme solution used for the digestion of tumour fragments consisted of RPMI-1640 with GlutaMAX, 25 mM HEPES pH 7.2, 100 U/ml penicillin, 100 μg/ml streptomycin, 1 mg/ml collagenase (Sigma-Aldrich) and 0.0125 mg/ml Pulmozyme (Roche).

### Manufacturing of TILs

Tumour tissue was isolated from the surrounding tissue and cut into 1–3 mm^3^ fragments with a scalpel. Fragments were extensively washed with phosphate-buffered saline (PBS) before plating to minimise the risk of contaminating TIL cultures with peripheral blood. Tumour fragments were plated in individual wells in a 24-well culture plate with 2 ml CM. Half the media was changed three times a week. TILs were harvested when pooled TIL micro-cultures generated from 48 fragments reached a total number of ≥100 × 10^6^ cells. This product, named Young TILs, had a generation time limit within 60 days. Young TILs were further expanded for 14 days in a small scale REP, as previously described.^19^ Briefly, 100,000 TILs were mixed with 20 × 10^6^ allogenic feeder cells, OKT3 antibody and master mix media containing 50% CM + 50% RM with 10% inactivated human AB serum. Young TILs generated in parallel from tumour specimens of metastatic melanoma (MM) were used as a comparison.

### Generation and validation of TCLs

TCLs were established either directly from tumour fragments, from media used for transportation of the tumour specimens or from enzymatically digested fresh tumour fragments, named fresh tumour digest (FTD). In addition, single-cell suspensions of uncultured FTDs containing all cells present in the tumour microenvironment (TME) were cryopreserved for later use.

TCLs were validated by (1) cytospin centrifugation of the cell suspension for morphologic evaluation and (2) formalin-fixation and paraffin-embedding (FFPE) followed by immunohistochemistry (IHC) staining for various OC markers: CA125, EpCAM, PAX8, p16, p53, CK7, the mesothelial cell marker Calretinin and the proliferation marker Ki67.

Selected TCLs were tested for expression of HLA class I and HLA class II using FACS analysis. The cells were stained with either panHLA-class I-APC, panHLA-class II-FITC or IgG1k APC and FITC isotypes (all from BD Bioscience, New Jersey, United States). All cells were stained with the 7ADD live/dead marker prior to analysis with a FACS Canto II instrument (BD Biosciences, New Jersey, United States) measuring median fluorescence intensity (MFI). The cutoff for positive expression was defined as 3× isotype MFI.

All cell lines were established internally from clinical material; thus, no testing for mycoplasma infection was done.

### Immunohistochemistry

Within 2 hours after surgery, the tumour specimens were cut into small fragments and several fragments were randomly picked for snap freeze samples in order to represent all parts of the tumour tissue. Five to 7 fragments were formalin fixed and embedded in paraffin before performing IHC. The samples were stained for cytokeratin A for localisation of the tumour tissue among stromal tissue. The samples were stained for the surface immune cell markers CD45, CD4, CD8, CD20 and CD56. The stained immune infiltrates were counted in three squares covering 0.201 mm^2^ each on every slide, using both NanoZoomer Digital Pathology (NDP.view 2) software and Fiji Image J 1.49 software. The three squares were placed on identical spots on every slide from the same patient with the aim to cover as much tumour tissue as possible. A consultant pathologist from Herlev Hospital supervised all the operations.

### Phenotyping of TILs

Both Young TILs and rapid expanded TILs (REP-TILs) were stained using fluorochrome-labelled monoclonal antibodies (mAb; from BD Bioscience, unless indicated otherwise) CD3-BV510, CD4-PerCP, CD8-BV421, CD45RA-FITC, CD45RO-PE, CCR7-PE-Cy7, CD62L-APC, CD69-PE-Cy7, CD28-APC, CD16-FITC (Dako, Glostrup, Denmark), CD137-PE, CD56-PE-Cy7, Gamma-Delta-TCR-PE (Biolegend, California, United States), LAG-3-FITC (LS Bioscience, Seattle, WA, United States), BTLA-PE, PD-1-PE-Cy7, TIM-3-APC (eBioscience, California, United States) and analysed with a FACS Canto II instrument (BD Biosciences). The phenotypic subpopulations was investigated for statistical difference using GraphPad Prism, Wilcoxon matched-pairs signed rank test.

### Evaluation of tumour reactivity

Antitumour reactivity of in vitro-expanded TILs was evaluated after co-culture for 5 hours of the TILs with autologous FTDs (thawed and washed twice) or autologous TCLs pretreated with interferon-γ (IFN-γ) (100 IU/ml), Peprotech, London, United Kingdom) or left untreated in a ratio of 3:1. Golgi plug and CD107a-BV421 were added at the beginning of incubation. Afterward, TILs were stained for the surface markers Near-IR Live/Dead (Life Technologies, California, United States), CD3-FITC, CD56-PE, CD8-QD605 (Life Technologies) and CD4-PerCP (Biolegend) and after overnight fixation and permeabilisation (eBioscience) further stained for intracellular cytokines TNF-APC and IFN-γ-PE-Cy7 (all chemicals and antibody mentioned in this paragraph are from BD bioscience unless indicated otherwise). The TILs were analysed with FACS Canto II (BD Bioscience). Tumour-specific TILs were defined as the frequency of T cells expressing at least one of the following T cell functions: tumour necrosis factor alpha (TNF-α), IFN-γ or CD107a. A specific antitumour response was defined as the presence of minimum 0.5% responding cells, with a minimum number of 50 positive events. The frequency of tumour-reactive cells in stimulated samples was subtracted from the unstimulated samples. 0.5% was used as a threshold for detection of tumour reactivity. All TIL products were tested for tumour reactivity against FTD except TILs from patient OC.TIL.03 and against autologous TCL (available from 11 patients).

### Cytotoxicity assays

A standard Cr^51^ cytotoxicity assay was performed as previously described.^20^ Briefly, 5 × 10^5^ tumour cells (target cells) were pulsed with 20 μl Cr^51^ for 1 hour. Autologous TILs (effector cells) and target cells were co-cultured for 4 hours and the Cr^51^ content of the supernatants was measured.

Results were further validated with an impedance-based assay (xCELLigence assay) for real-time detection of cytotoxicity of adherent cells.^21^ Tumour cells were added to the wells, 20,000 cells per well. After 2 days, when tumour cells reached a confluent stage, TILs were added with a different effector:target ratios. HLA-blocking antibodies (W6/32 and Tü39 Biolegend) were added 30 min prior to the TILs with final concentration 20 µg/ml. The tumour killing was monitored in real time, with the xCELLigence instrument (ACEA Bioscience, San Diego, CA, United States).

### Immunopurification of HLA class I peptides

Immunopurification of HLA class I peptides was performed on a plate format using a positive pressure processor (Waters, Milford, Massachusetts), as previously described.^22^ Briefly, frozen cell pellets of OC.TIL.11 (1.6 × 10^8^ cells) were lysed for 1 hour at 4 °C with PBS containing 0.25% sodium deoxycholate (Sigma-Aldrich), 0.2 mM iodoacetamide (Sigma-Aldrich), 1 mM EDTA, 1:200 Protease Inhibitors Cocktail (Sigma-Aldrich), 1 mM phenylmethylsulfonylfluoride (Roche), 1% octyl-beta-D glucopyranoside (Sigma-Alrich). Lysates were cleared by centrifugation (Eppendorf Centrifuge, Hamburg, Germany) at 4 °C at maximum speed for 50 min. The plate’s wells were equilibrated before the addition of anti-panHLA-I (W6/32) antibodies covalently cross-linked to Protein-A Sepharose beads (Invitrogen, California, USA). The lysates were passed through the wells by gravity flow at 4 °C. Thereafter, washes were performed with four column volumes sequentially of 150 mM, 400 nM and lastly again 150 mM in 20 mM Tris-HCl pH 8, using the processor. Lastly, beads were washed with two column volumes of 20 mM Tris-HCl pH 8.

HLA-I peptides were purified with the application of a Sep-Pak tC_18_ 96-well plate (Waters). The Sep-Pak plate was first conditioned with 80% acetonitrile (ACN, Merck) in 0.1% trifluoroacetic acid (TFA, Merck, Darmstadt, Switzerland) and subsequently also with 0.1% TFA. HLA complexes and the bound peptides were directly eluted on to the Sep-Pak plate with 500 µl 1% TFA. C_18_ wells were then washed with 0.1% TFA before elution of HLA-I peptides with 28% ACN in 0.1% TFA. Recovered HLA-I peptides were dried using vacuum centrifugation (Thermo Fisher Scientific).

### Liquid chromatography-mass spectrometry analysis

Liquid chromatography-mass spectrometry (LC-MS) analysis was performed with the nanoflow Ultra-HPLC Easy nLC 1200 (Thermo Fisher Scientific, LC140) coupled online to a Q Exactive HF Orbitrap mass spectrometer (Thermo Fischer Scientific) with a nanoelectrospray ion source (Sonation, PRSO-V1; Baden-Württemberg, Germany) as previously described.^22^ Peptides were eluted over 125 min with a gradient of 0.1% FA in 80% ACN. Data were acquired with a data-dependent method and HCD fragmentation at a normalised collision energy of 27%. The MS scan range was set to 300 to 1650*m*/*z* with a resolution of 60,000 (200*m*/*z*) and an AGC target value of 3 x 10^6^ ions. For MS/MS, AGC target values of 1 x 10^5^ were used with a maximum injection time of 120 ms at a set resolution of 15,000 (200*m/z*). In case of unassigned precursor charge states, or charge states of four and above, no fragmentation was performed. The dynamic exclusion of precursor ions from further selection was set for 20 s.

### Identification of tumour-associated HLA-binding peptides

We employed the MaxQuant computational proteomics platform version 1.5.5.1 to search the peak lists against the UniProt databases (Human, 42,148 entries, March 2017) and a file containing 247 frequently observed contaminants. Methionine oxidation (15.994915 Da) was set as variable modification. The second peptide identification option in Andromeda was enabled. An FDR of 0.05 and no protein FDR was set with unspecific enzyme specificity. Possible sequence matches were restricted to 8–25 amino acids. The initial allowed mass deviation of the precursor ion was set to 6 ppm and the maximum fragment mass deviation was set to 20 ppm. We enabled the “match between runs” option, which allows matching of identifications same biological samples in a time window of 0.5 min and an initial alignment time window of 20 min. We extracted the “peptides” MaxQuant output table and filtered out peptides matching to reverse and contaminants. For in vitro validation of immunogenicity, we selected 23 HLA peptides (Supplementary Table 2) derived from known tumour-associated proteins based on literature and tumour specificity.

### Evaluation of peptide recognition

Screening for peptide recognition was carried out with IFN-γ ELISPOT assays. Briefly, nitrocellulose bottomed 96-well plates (Merck Millipore, Søborg, Denmark) were coated overnight at room temperature (RT) with anti-IFN-γ antibody 1-D1K (Mabtech, Nacka Strand, Sweden). The plates were washed six times in PBS and blocked by X-vivo medium (Lonza, Basel, Switzerland). The TILs were added in triplicates, 100,000 cells/well. The selected peptides (Supplementary Table 2) was added with a final concentration of 5 µM alongside a negative control without peptide and a positive control (*Staphylococcus* Enterotoxin B (SEB)). After overnight incubation at 37 °C and 5% CO_2_, the plates were washed in PBS and IFN-γ biotinylated secondary Ab (Mabtech) was added followed by a further 2 hours incubation at RT. The plates were then again washed with PBS and Streptavidin-ALP (Mabtech) was added followed by 1 hour incubation at RT. Finally, the plates were washed and enzyme substrate NBT/BCIP (Mabtech) was added. The spots were counted using the ImmunoSpot Series 2.0 Analyzer (CTL Analyzer, Bonn, Germany). Background spots were subtracted from the peptide spots and an ELISPOT response was defined as more than 20 spots after background subtraction.

Anti-GAGE reactivity of in vitro-expanded TILs was evaluated as previously described in the section Evaluation of tumour reactivity, though the TILs were incubated with peptides (final concentration of 10 µM) for 7 hours. The TILs were analysed with FACS Canto II (BD Bioscience).

### HLA tetramer staining and cell sorting

Tetramers coupled with PE and APC were prepared in-house, as described previously.^23^ REP-TILs were stained with CD8-PerCP, CD4-FITC (both BD Bioscience), NIR and the HLA tetramer complex HLA-A3/GAGE-peptide conjugated with PE/APC. Tetramer-positive cells were sorted using FACS Aria (BD Bioscience, New Jersey, United States) and immediately expanded with allogeneic irradiated PBMCs, human serum and IL-2 (two consecutive REP procedures at smaller scale were carried out). In addition, GAGE-specific clones were prepared by sorting one cell/well in a round bottom 96-well plate containing allogeneic irradiated PBMCs, human serum and IL-2 as described above.

## Results

### Processing of specimens, initial TIL outgrowth and TCL generation

Characteristics of the clinical specimens are reported in Table 1. All patients included in this study had histologically confirmed OC, the vast majority at an advanced disease stage (FIGO III or IV). Various ovarian tumour histologies were represented, including rare carcinosarcomas. Supplementary Figure 1A illustrates the distribution of histologic subtypes in this cohort (*n* = 33).

At our centre, we have considerable experience in manufacturing of TILs from MM,^19,24^ head and neck cancer^25^ and primary renal cell carcinoma (pRCC).^26^ In general, resected tumour specimens, from MM and pRCC, contains a well-defined area with tumour tissue which can easily be dissected from surrounding healthy tissues. In this study, all samples from OC were obtained from intraperitoneal metastases. In contrast to melanoma and pRCC, these samples were highly heterogeneous, typically more difficult to dissect from surrounding healthy tissues and in some cases heavily infiltrated with mucinous areas (data not shown). Young TIL cultures were successfully established from all samples within 60 days (median 28 days, range [15–59]).

Autologous TCLs were established from 11 out of the 34 OC specimens, including from subtypes such as serous adenocarcinoma, carcinosarcoma and clear cell adenocarcinoma. TCLs were established most successfully from transport media (7/34 cases). Establishment of TCLs from fragments and ascites were successful in 3/34 and 1/11 cases, respectively. From one patient TCLs were established from both the fragments and the transport media. The distribution of the subtypes of these TCLs is illustrated in Supplementary Figure 1B.

### Massive expansion (rapid expansion) of TILs

The REP is currently used in clinical trials of adoptive cell transfer (ACT) to obtain large numbers of autologous TILs for intravenous infusion.

**Fig. 1:**
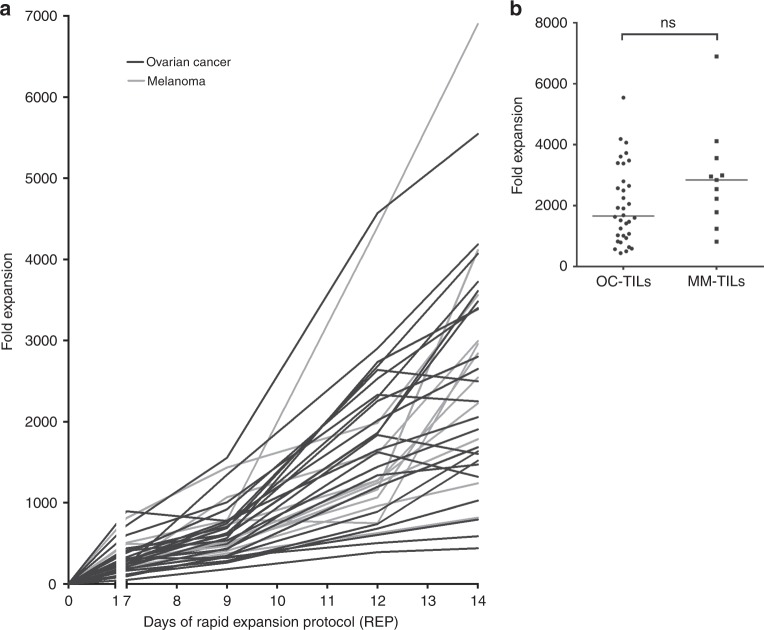
Ovarian tumour-infiltrating lymphocytes (TILs). Young TILs were further expanded using a small scale Rapid Expansion Protocol (REP). **a** Fold expansion of TILs from 24 ovarian cancer (OC) patients (purple lines) and from 11 metastatic melanoma (MM) patients (blue lines) following a 14 days REP performed in parallel were compared. Median fold expansion for all OC patients (*n* = 34) was 1660 (range [440–5544]). Median fold expansion for MM (*n* = 11) was 2842 (range [816–6900]). **b** Scatterplot showing the final fold expansion. No significant difference was seen when comparing the final fold expansion of OC-TILs (*n* = 34) and MM-TILs (*n* = 11). The two groups were compared using the Mann–Whitney test. Data are presented with the median

All Young TIL cultures were further expanded with a median fold expansion of more than 1500-fold (median 1660, range [440–5544); Fig. 1a). Growth kinetics and fold expansions were compared head-to-head to MM (*n* = 11). Median fold expansion in MM-TIL appeared higher, median 2842 (range [816–6900]; *p* = 0.09 compared to OC-TIL); however, the difference was not statistically significant (Fig. 1b). In addition, a comparison of the fold expansion of TILs from chemotherapy naïve patients (*n* = 20) and TILs from patients treated with chemotherapy (*n* = 14) showed no significant difference (data not shown).

### Phenotype of TILs

**Fig. 2:**
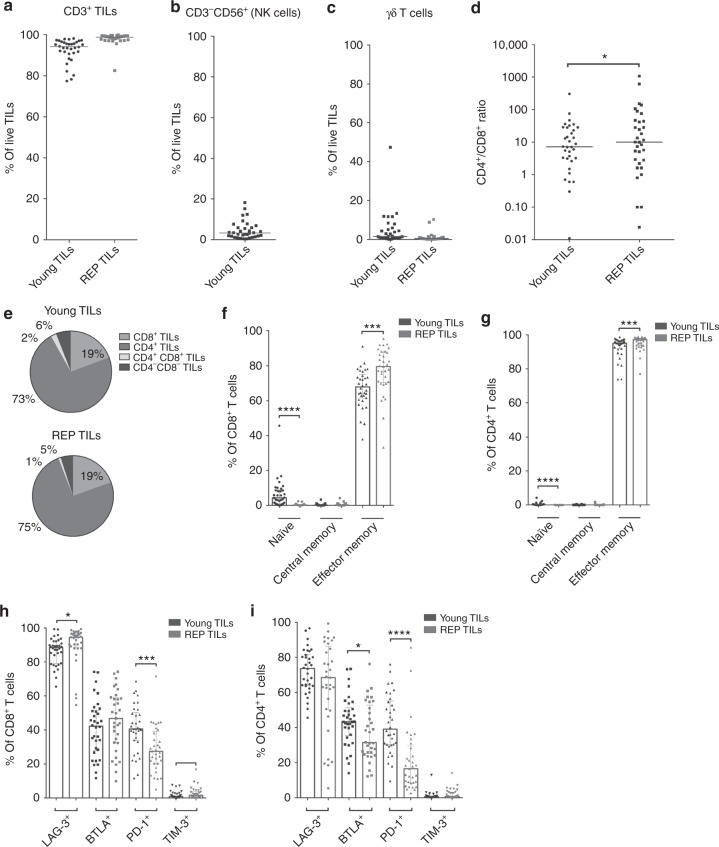
Phenotypic characterisation of ovarian cancer TILs. Young TILs and REP-TILs were analysed with flow cytometry for phenotypic markers. **a** Scatterplot showing percentages of CD3^+^ T cells in Young TIL (*n* = 34) and REP TIL (*n* = 33) populations. Data are presented with the median. **b** Scatterplot showing the percentage of NK cells in the Young TIL population (*n* = 34). Data are presented with the median. **c** Scatterplot showing percentages of γδ T cells in Young TIL (*n* = 34) and REP TIL (*n* = 33) populations. Data are presented with the median. **d** Scatterplot showing the CD4/CD8 ratios in Young TILs (*n* = 34) and REP-TILs (*n* = 33). The median CD4/CD8 ratio in Young TILs was 7.2 (range [0.01–306]) and 10.0 (range [0.02–1067]) in REP TIL population. Data were log-transformed before the two groups were compared. A significant increase in the ratio was observed (*p* = 0.0253). Data are presented with the median. **e** The pie charts show the phenotypic distribution of CD8^+^, CD4^+^, CD4^+^CD8^+^ and CD4^−^CD8^−^ of CD3^+^ TILs in the Young TIL (*n* = 34) and REP TIL (*n* = 33) populations. Data are presented with mean values. **f**, **g** Scatterplots illustrating the percentage of naïve T cells, central memory T cells and effector memory T cells and **h**, **i** Scatterplots illustrating the percentage of Exhaustion markers: LAG-3, BTLA, PD-1 and TIM-3 in **f**, **h** the CD8^+^ T cell population and **g**, **i** the CD4^+^ T cell population in Young TILs (*n* = 34) and REP-TILs (*n* = 33). Data are presented with the median with interquartile range. Panel **f** shows a significant increase of CD8^+^ Tem cells *p* = 0.0004 during the REP and a significant decrease in the Naïve T cell subpopulation, *p* < 0.0001. **g** The CD4-expressing population had a significant increase in CD4^+^ Tem cells during the REP, *p* = 0.0003, and a significant decrease in the Naïve T cell subpopulation during the REP, *p* < 0.0001. Panel **h** shows significant increase of CD8^+^ LAG-3^+^ T cells *p* = 0.0263 during the REP and a significant decrease in the PD-1^+^ T cells, *p* = 0.0002, and a significant increase in the TIM-3^+^ T cells, *p* = 0.0379. **i** The CD4-expressing population had a significant decrease in CD4^+^ BTLA^+^ cells during the REP, *p* = 0.013, and a significant decrease in the PD-1^+^ T cell subpopulation during the REP, *p* < 0.0001. Young TILs and REP-TILs were compared using the Wilcoxon signed rank test. Statistical significant differences is indicated with *, **, *** or **** for *p* values less than 0.05, 0.01, 0.001, or 0.0001, respectively

Young TILs contained mainly CD3^+^ cells (median 94.2%, range [77.4–98.2], Fig. 2a), with a larger proportion of CD4^+^ T cells than CD8^+^ T cells. A low frequency of NK cells (defined as CD3^−^CD56^+^ lymphocytes) was detected in most patients (median 3.3% of lymphocytes, range [0.3–18.2]; Fig. 2b). In addition, we also found a small proportion of γδ T cells (median 1.5%, range [0.1–47.4] (Fig. 2c).

The CD4/CD8 ratio was highly variable among all 33 individual patients (median 7.2, range [0.01–306]; Figs. 2d, e). Most CD4^+^ and CD8^+^ T cells displayed a phenotype consistent with experienced effector memory T cells (Tem; CD45RO^+^CCR7^−^CD62L^−^), as defined by Sallusto et al.^27^

REP-TILs contained almost exclusively CD3^+^ T cells, with a slightly higher CD4/CD8 ratio (10, range [0.02–1067], *p* = 0.07 vs Young TILs, Fig. 2d). After REP, the fraction of CD8^+^ Tem cells increased (*p* = 0.0004) along with a reduced fraction of Naïve CD8^+^ T cells (*p* < 0.0001). The same pattern was observed in the CD4^+^ T cell subpopulation Tem cells increased (*p* = 0.0003) along with a reduced fraction of Naïve T cells (*p* < 0.0001)) (Figs. 2f, g).

Regarding the classic exhaustion markers, a decrease in the PD-1^+^ cells was observed in both the CD8^+^ and CD4^+^ subpopulations after REP (*p* = 0.0002 and *p* < 0.0001, respectively). A relative high fraction of LAG-3^+^ CD8^+^ CD4^+^ TILs were observed, Young TILs CD8^+^ and CD4^+^ median 88.5% range [65.5–99.3] and median 73.7% range [45.6–96.6], respectively, and REP-TILs CD8^+^ and CD4^+^ median range and median range, respectively. Meanwhile, a significant increase of the LAG-3^+^ CD8^+^ subpopulation after REP was observed (*p* = 0.0263) (Figs. 2h, i). In addition, the expression of three markers, CD69, CD137 and CD28 was determined (Supplementary Figure 2A and 2B).

In order to determine whether TILs were truly expanded from the TME, the characteristics of the immune infiltrate in situ was further analysed and compared to expanded TILs from five specimens. Analysis of immune infiltrates in situ showed high inter-patient variations (representative examples see Supplementary Figure 3). The distribution of the four immune markers analysed (CD4, CD8, CD20 and CD56) is illustrated in Supplementary Figure 4A. The distribution of the four immune markers was similar in FTD (Supplementary Figure 4A). The distribution of CD4^+^ and CD8^+^ T cells in the TME (IHC data) was compared to the distribution of the expanded CD4^+^ and CD8^+^ TILs from the same specimens (Supplementary Figure 4B). In TILs from each individual specimen, it appeared that similar CD4 to CD8 ratios were maintained throughout TIL expansion, with a tendency of an increase in CD4^+^ T cells (Supplementary Figure 4B). Overall, these data suggest that TILs are indeed expanded from the TME of OC and not from surrounding healthy tissues.

### Tumour reactivity

**Fig. 3:**
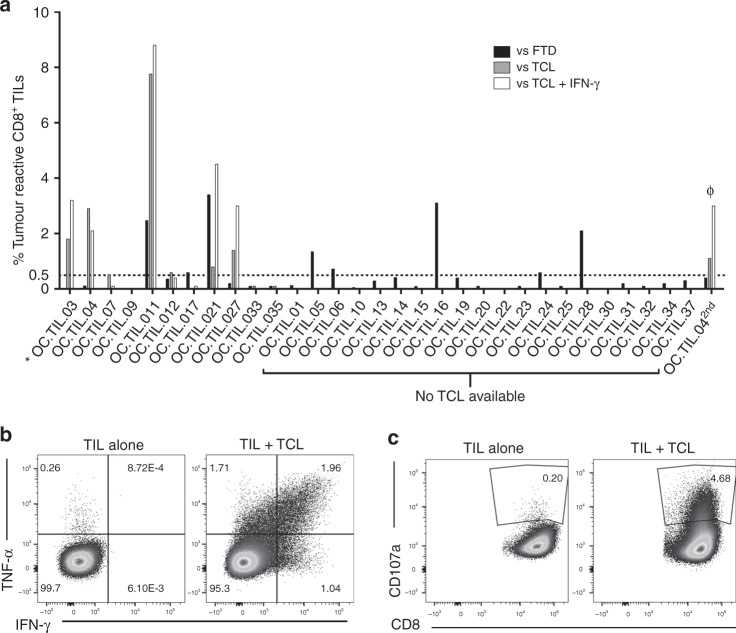
In vitro antitumour activity of CD8^+^ Young TILs. The antitumour activity of the in vitro-expanded TILs was evaluated by defining the frequency of T cells expressing at least one of the following T cell functions: TNF-α, IFN-γ or CD107a, upon stimulation with autologous Fresh tumour digest (FTD) or tumour cell line (TCL) treated with low-dose IFN-γ (100 IU/ml) or left untreated. A specific antitumour response was defined as the presence of minimum 0.5% responding cells, with a minimum number of 50 positive events. The frequency of tumour-reactive cells in stimulated samples was subtracted from the unstimulated samples. In all, 0.5% was used as a threshold for detection of tumour reactivity. **a** Antitumour responses of CD8^+^ T cells were detected in 13 of 31 patients analysed. *: OC.TIL.03 is not tested with FTD. ϕ: TILs generated from OC.TIL.04 2^nd^ was tested for reactivity against FTD from OC.TIL.04. **b** FACS plot showing cytokine production from TIL alone (unstimulated, serving as a negative control) and TIL stimulated with autologous TCL, from a representative patient (OC.TIL.11). **c** FACS plot showing CD107a mobilisation of TIL upon co-culture with an autologous TCL. Unstimulated TIL (TIL alone) serves as a negative control. An example of the gating strategy is showed in Supplementary Figure 7A

The antitumour activity of the in vitro-expanded TILs was evaluated after co-culture of TILs and either autologous FTD or TCLs. Responses of CD8^+^ T cells towards autologous TCLs were detected in 7 of 11 patients, the median frequency in responders 1.4%, range [0.51–7.8]. Responses towards autologous TCLs pretreated with IFN-γ were detected in 5 of 11 patients, median frequency in responders 3.2%, range [2.1–8.8]. Antitumour responses of CD8^+^ T cells to autologous FTD was observed in 8 of 30 patients tested, and the median frequency in responders was 1.7%, range [0.6–3.4]. In total, CD8^+^ T cell responses against autologous tumour cells were detected in 13 out of 31 patients tested, with a median frequency of 3.05% (considering only the highest value, when different assays were available for one individual patient) (Fig. 3).

**Fig. 4:**
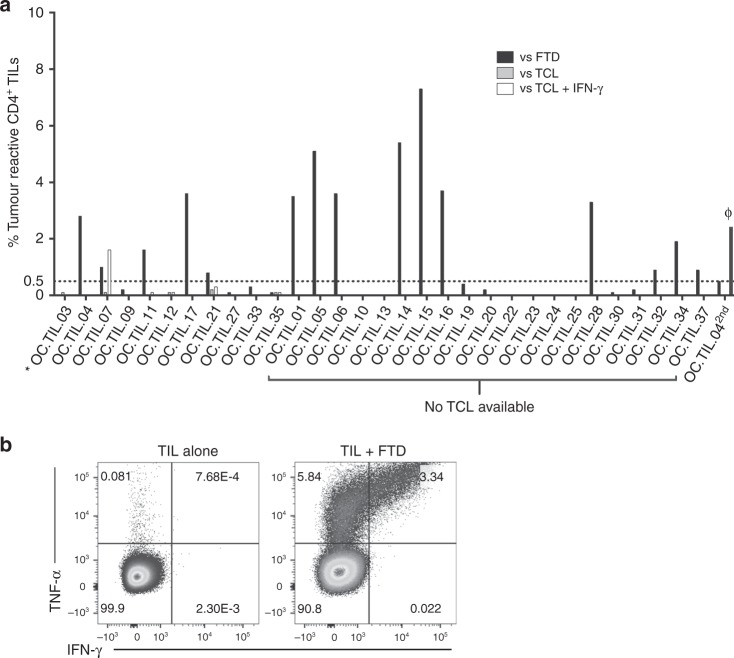
In vitro antitumour activity of CD4^+^ Young TILs. The antitumour activity of the in vitro-expanded TILs was evaluated by defining the frequency of T cells expressing at least one of the following T cell functions: TNF-α, IFN-γ or CD107a, upon stimulation with autologous fresh tumour digest (FTD) or tumour cell line (TCL) treated with low-dose IFN-γ (100 IU/ml) or left untreated. A specific antitumour response was defined as the presence of minimum 0.5% responding cells, with a minimum number of 50 positive events. The frequency of tumour-reactive cells in stimulated samples was subtracted from unstimulated samples; 0.5% was used as a threshold for detection of tumour reactivity. **a** Antitumour responses of CD4+ T cells were detected in 16 of 31 patients. *OC.TIL.03 is not tested with FTD. ϕ: TILs generated from OC.TIL.04 2^nd^ was tested for reactivity against FTD from OC.TIL.04. **b** FACS plot showing cytokine production from TIL alone (unstimulated, serving as a negative control) and TIL stimulated with autologous TCL, from a representative patient (OC.TIL.15). An example of the gating strategy is showed in Supplementary Figure 7A

Responses of CD4^+^ T cells to autologous TCLs were only detected when the TCL had been pretreated with IFN-γ and in this case, a response of 1.6% of the CD4^+^ T cells was only detected in 1 of 11 patients (OC.TIL.07; 9%). Six selected TCLs (OC.TIL.03, -04, -07, -09, -11, -33) were analysed for HLA class I and HLA class II expression. All TCLs expressed constitutively HLA class I. None of the TCL expressed HLA class II constitutively, but treatment with IFN-γ induced high or moderate HLA class II expression in three cell lines (OC.TIL.07, -09, -33; Supplementary Figure 5). Antitumour responses of CD4^+^ T cells to autologous FTD were detected in 15 of 30 patients (50%), median frequency in responders 3.3%, range [0.8–7.3]. In total, CD4^+^ T cell responses to autologous tumour cells were detected in 16 out of 31 patients tested (52%), with a median frequency of 3.3% (considering only the highest value, when different assays were available for one individual patient) (Fig. 4).

Overall, antitumour T cell responses (either CD8^+^ or CD4^+^ T cells) were detected in 19 of 31 patients tested (61%)

We observed an NK cell antitumour response in 8 of 29 patients. Although the median frequency of responding NK cells was generally low (1.45%), occasionally a high fraction of NK cells recognised autologous TCLs (Supplementary Figure 6). The NK cell antitumour responses were found in both TILs with and without an in vitro antitumour T cell response (data not shown).

We determined whether TILs from selected patients with OC had cytolytic activity to autologous tumours. Young TILs from patient OC.TIL.11 generated highly specific lysis of the autologous tumour cells; however, the cytotoxic potential of the TILs decreased after the TILs have been rapid expanded (Supplementary Figure 7A). Young TILs from patient OC.TIL.04 also generated specific lysis of the autologous tumour cells, whereas lysis capacity disappeared after REP (Supplementary Figure 7B). For patient OC.TIL.11, the cytolytic potential of TILs was confirmed with another assay (xCELLigence assay), where the T cell-tumour interplay can be studied in real time for a prolonged period. A clear correlation between the number of effector cells per target cells and the duration of time before complete tumour elimination was observed (Supplementary Figure 7C).

### Identification of immunogenic antigens presented on OC cells

**Fig. 5:**
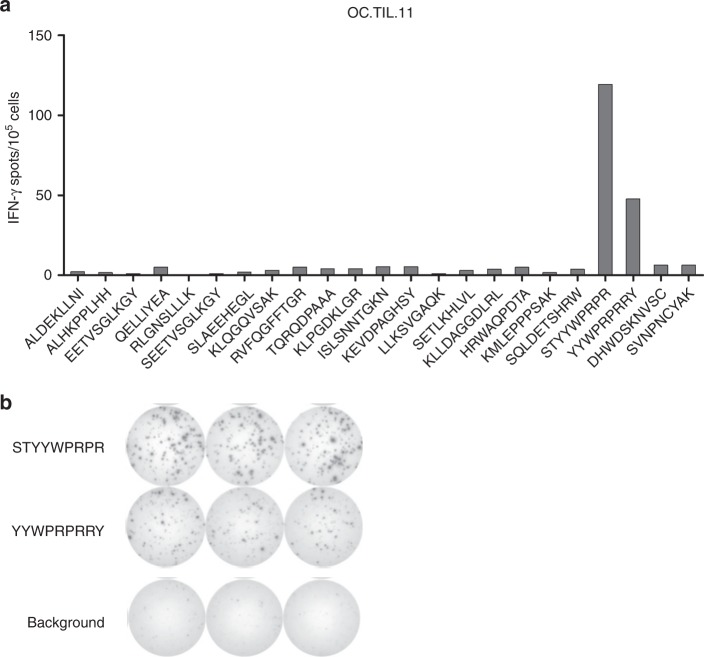
IFN-γ ELISPOT assay: Selected peptides identified by MS-based immunopeptidomics tested for the ability to induce an IFN-γ response in REP-TILs from patient OC.TIL.11. Background (spots in wells without added peptides) was subtracted. Example of IFN-γ ELISPOT response against GAGE peptides

One OC cell line (OC.TIL.11) was analysed with LC-MS-based immunopeptidomics and multiple tumour-associated antigens were identified. The immunogenicity of these peptides was determined using the IFN-γ ELISPOT assay. We identified two overlapping peptides of the GAGE cancer-testis antigen family STYYWPRPR and YYWPRPRRY eliciting a clear IFN-γ response (119 and 47 spots, respectively) in patient OC.TIL.11 (Fig. 5). The responses in OC.TIL.11 were confirmed with ICS and flow cytometry analysis (Supplementary Figure 8).

**Fig. 6:**
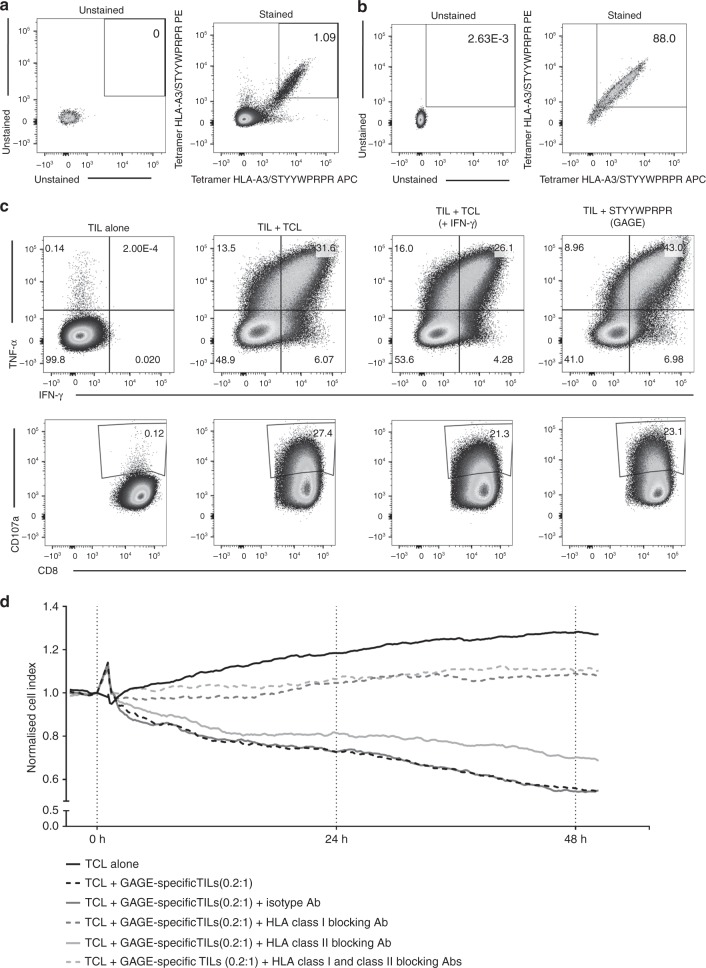
GAGE-specific TILs. **a** 1600 GAGE12-specific Young TILs from patient OC.TIL.11 were sorted using tetramers HLA-A3/STYYWPRPR APC/PE. **b** The sorted culture was tested for specificity with tetramers HLA-A3/STYYWPRPR APC/PE after two times REP. **c** FACS plot illustrating cytokine production (upper panel) and CD107a mobilisation (lower panel) in TIL alone (unstimulated) as a negative control, TILs stimulated with autologous tumour cell line (TCL), TILs stimulated with autologous TCL pre-stimulated with IFN-γ for 3 days prior to experiment and TILs stimulated with the GAGE peptide. An example of the gating strategy is showed in Supplementary Figure 7A. **d** xCELLigence killing assay showing the killing of autologous tumour cells with GAGE-specific TILs from patients OC.TIL.11 (ratio 0.2:1). HLA class I antibody, HLA class II antibody and a combination were added as controls

GAGE-specific TILs were sorted using tetramer complexes of HLA-A3/STYYWPRPR and further expanded with two consecutive REPs. This procedure resulted in one T cell culture highly enriched with GAGE-specific T cells (around 90% stained positive for the HLA-A3/STYYWPRPR-tetramer). This GAGE-specific T cell-enriched culture displayed a very high recognition of both the GAGE-peptide STYYWPRPR and autologous TCL (Fig. 6). Furthermore, the GAGE-specific culture’s ability to kill autologous tumour cells was confirmed with the xCelligence assay. The addition of HLA class I and HLA class II antibodies further confirmed that the response is HLA class I dependent (Fig. 6c). Similar high immune recognition was observed with one GAGE-specific clone obtained from the same original TILs (Supplementary Figure 9).

## Discussion

Recent successes of ACT with TILs in MM warrant investigating this treatment strategy in other cancer types. Until recent years, current TIL manufacturing methods had not been applied to other immunogenic solid tumours such as OC.

In this study, TILs from all patients were expanded to sufficient numbers for clinical application. OC-TILs displayed growth properties similar to those of MM-TILs. We expanded TILs from MM specimens to reach $\sim$100 × 10^9^ cells (5000 fold expansion) for infusion^28^ and clinical responses were achieved in over 40% of treated patients.^24^

We investigated the phenotype of the TILs before and after the REP with a broad panel of surface markers and found an abundance of effector memory T cells. This is consistent with phenotype analyses performed in other solid tumours, e.g., MM.^19^ The distribution of major T cell subpopulations, including CD4^+^ and CD8^+^ T cells, did not change dramatically during the REP. In general, we found a higher frequency of CD4^+^ T cells than CD8^+^ T cells in the expanded OC-TIL products, which was also true in the original TME. This finding differs from previous observations in the majority of melanomas.^29^ In addition, we observed a relatively high expression of the exhaustion marker LAG-3 in the TILs, which could be induced by high concentration of IL-2 in the culture medium and it could be a piece in the puzzle of the low antitumour reactivity in TILs.

A recent study by the Brad Nelson group^30^ showed that OC patients receiving neoadjuvant chemotherapy had tumour tissue with increased densities of CD3^+^ and CD8^+^ T cells compared to chemotherapy naïve tumour tissue using IHC analysis. The tumour specimens in our study included 20 chemotherapy naïve specimens and 14 specimens from patients who received chemotherapy prior to the tumour resection. Our preliminary analysis of these two groups did not show dramatic differences in TIL expansion or phenotype (data not shown).

Current methods of TIL isolation require dissection of viable tumours from surrounding tissues. This is typically achieved by scalpel separation of the tumour tissue from the healthy tissue. Our experience and macroscopic characteristics of most OC tumours indicate that the scalpel separation technique is more difficult to apply on OC tumours than MM. It is of great importance that the lymphocytes are expanded from the TME and not from healthy tissue. By comparing the composition of in situ tumour infiltrates and expanded TILs, we provide indirect evidence that the TILs in our study were truly expanded from the TME.

Most importantly, we showed that tumour-reactive TILs could be recovered from the TME in the majority of patients with unselected histologies of OC. This observation resembles the findings in melanoma^31,32^ and other solid cancer types such as head and neck squamous cell carcinoma,^25^ metastatic gastrointestinal adenocarcinomas,^33^ and RCC.^26,34,35^ However, it appeared that the frequency of tumour-reactive T cells in OC-TILs was lower than reported in MM-TILs.^20^ A very recent study in OC with a slightly different manufacturing approach showed similar results as present findings, though with a markedly lower TIL expansion rate.^36^

In-depth phenotypic characterisation of immune responses showed that tumour-reactive CD4^+^ T cells frequently infiltrate OC. In one patient, we confirmed the direct recognition of autologous tumour cells by tumour-specific CD4^+^ T cells. In addition, we observed significantly more CD4^+^ T cell responses after co-culture with FTD than when using autologous TCLs. This can potentially be explained by the additional presence of antigen-presenting cells in the FTD, which can activate CD4^+^ T cells and thereby elicit greater immune responses compared to autologous TCLs. We and others have previously shown that CD4^+^ T cells can recognise autologous tumour, including mutant neo-antigens, presented directly by MM tumour cells.^20,37,38^ Based on these current findings, it appears that CD4^+^ T cells also play a role in the surveillance of the TME of OC.

Finally, we identified using advanced MS-based immunopeptidomics analysis tumour antigens directly presented on the tumour cells. We confirmed that two cancer-testis antigens from the GAGE family elicited T cell responses in vitro in one OC patient. The immunogenicity of one of these peptides (YYWPRPRRY) has already been reported previously.^39^ However, for the first time to our knowledge, GAGE-specific TILs to HLA peptide STYYWPRPR were successfully isolated and expanded. These findings are in-line with a recent study showing the presence of neo-epitope specific CD8^+^ TILs from OC.^40^ This specific population displayed a profound increase in the T cell recognition of the autologous tumour cells. However, Wick et al.^8^ showed that despite successful T cell recognition of neo-antigens in OC, this might not necessarily prevent tumour progression. The same group recently showed that the expression of neo-antigens in OCs is deficient compared to other cancer types. They only found a few neo-antigen-specific CD4^+^ and CD8^+^ T cell responses in vitro and none in vivo using neo-antigen-specific vaccines. OC is mostly driven by large-scale mutations, and current bioinformatic prediction tools may not be able to predict which neo-antigens are presented in OC.^41^ Additional methods, such as LC-MS-based immunopeptidomics, may provide additional insights into the antigen repertoire of OC.^42^

In conclusion, we show that TILs can efficiently be expanded from OC. However, unselected TILs from OC generally contain small amounts of tumour-reactive T cells. One of the limiting factors could be the ability to enrich for tumour-reactive T cells, which can be achieved using novel technologies.^9,40,43^ These data encourage further studies to test the efficacy of ACT in OC and highlight the need for improved methods of manufacturing to increase the quality of TIL products.

## Supplementary information

Supplementary Figure 9

Supplementary Tables

Supplementary Figure 1

Supplementary Figure 2

Supplementary Figure 3

Supplementary Figure 4

Supplementary Figure 5

Supplementary Figure 6

Supplementary Figure 7

Supplementary Figure 8

## Data Availability

Data and material generated and analysed during the current study can be available upon reasonable request to the corresponding author.
