# Correction: Tumour-reactive T cell subsets in the microenvironment of ovarian cancer

**DOI:** 10.1038/s41416-019-0425-6

**Published:** 2019-03-19

**Authors:** Marie Christine Wulff Westergaard, Rikke Andersen, Chloé Chong, Julie Westerlin Kjeldsen, Magnus Pedersen, Christina Friese, Thomas Hasselager, Henrik Lajer, George Coukos, Michal Bassani-Sternberg, Marco Donia, Inge Marie Svane

**Affiliations:** 1 Center for Cancer Immune Therapy, Department of Hematology, Herlev Hospital, University of Copenhagen, Copenhagen, Denmark; 2 Department of Oncology, Herlev Hospital, University of Copenhagen, Copenhagen, Denmark; 3 Ludwig Institute for Cancer Research, University of Lausanne, Lausanne, Switzerland; 4 Department of Oncology, University Hospital of Lausanne, Lausanne, Switzerland; 5 Department of Pathology, Herlev Hospital, University of Copenhagen, Copenhagen, Denmark; 6 Department of Gynaecology, Rigshospitalet, University of Copenhagen, Copenhagen, Denmark

**Keywords:** Tumour immunology, Ovarian cancer, Ovarian cancer, Tumour immunology

**Correction to:** *British Journal of Cancer* (2019) **120**, 424–434; 10.1038/s41416-019-0384-y; http://bjcancer.com;  published online 5 February 2019.

Since the publication of this paper, the authors noticed that the funding information was not complete. The correct funding information is now shown in the Acknowledgements section.

