# Supplementary figures and images for: Tumour-reactive T cell subsets in the microenvironment of ovarian cancer
Source: Br J Cancer. 2019 Feb 5;120(4):424–34. doi: 10.1038/s41416-019-0384-y (PMC6461863; doi:10.1038/s41416-019-0384-y)

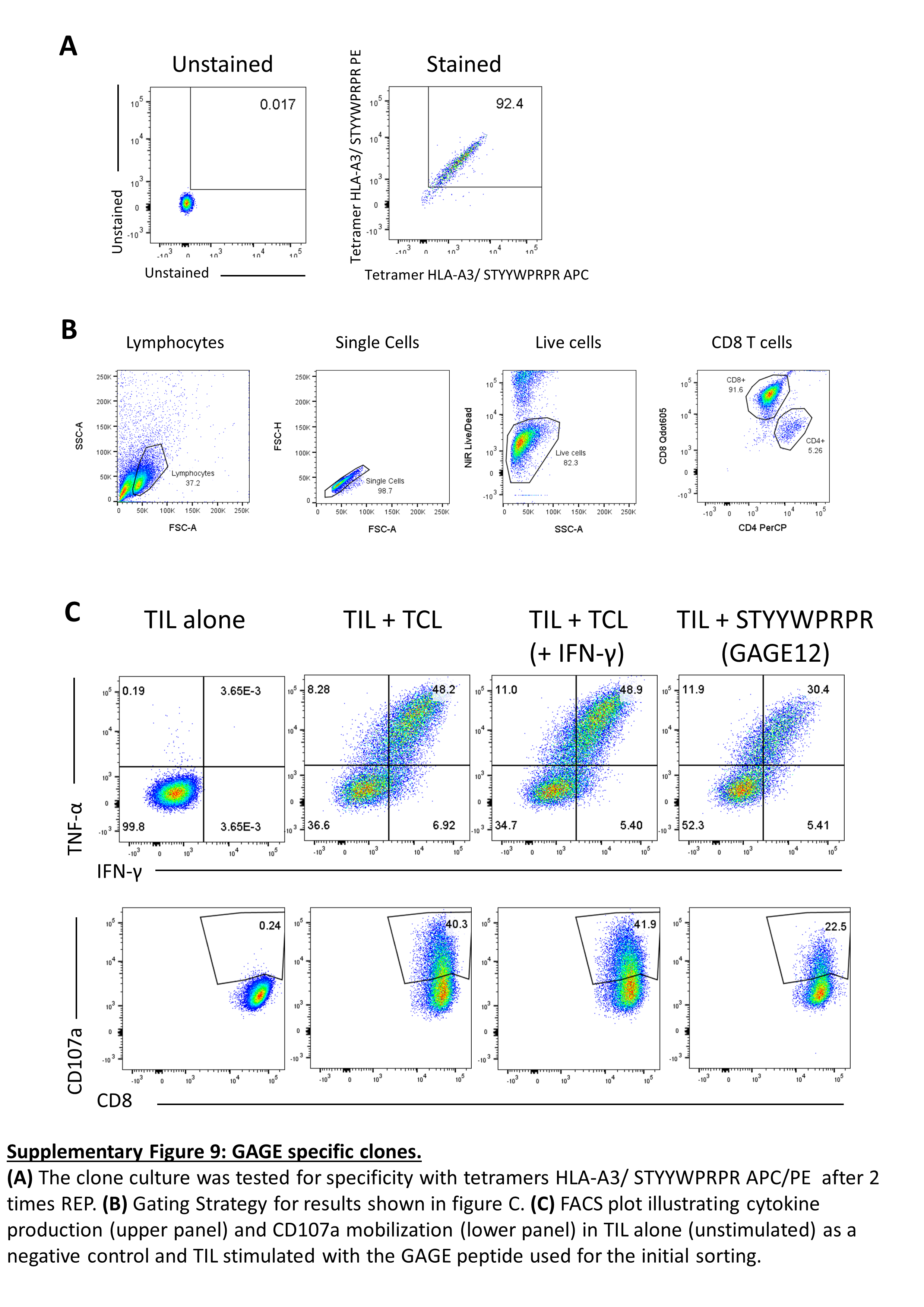

Supplement: Supplementary file 1 — Supplementary Figure 9 [file 41416_2019_384_MOESM1_ESM.tif]

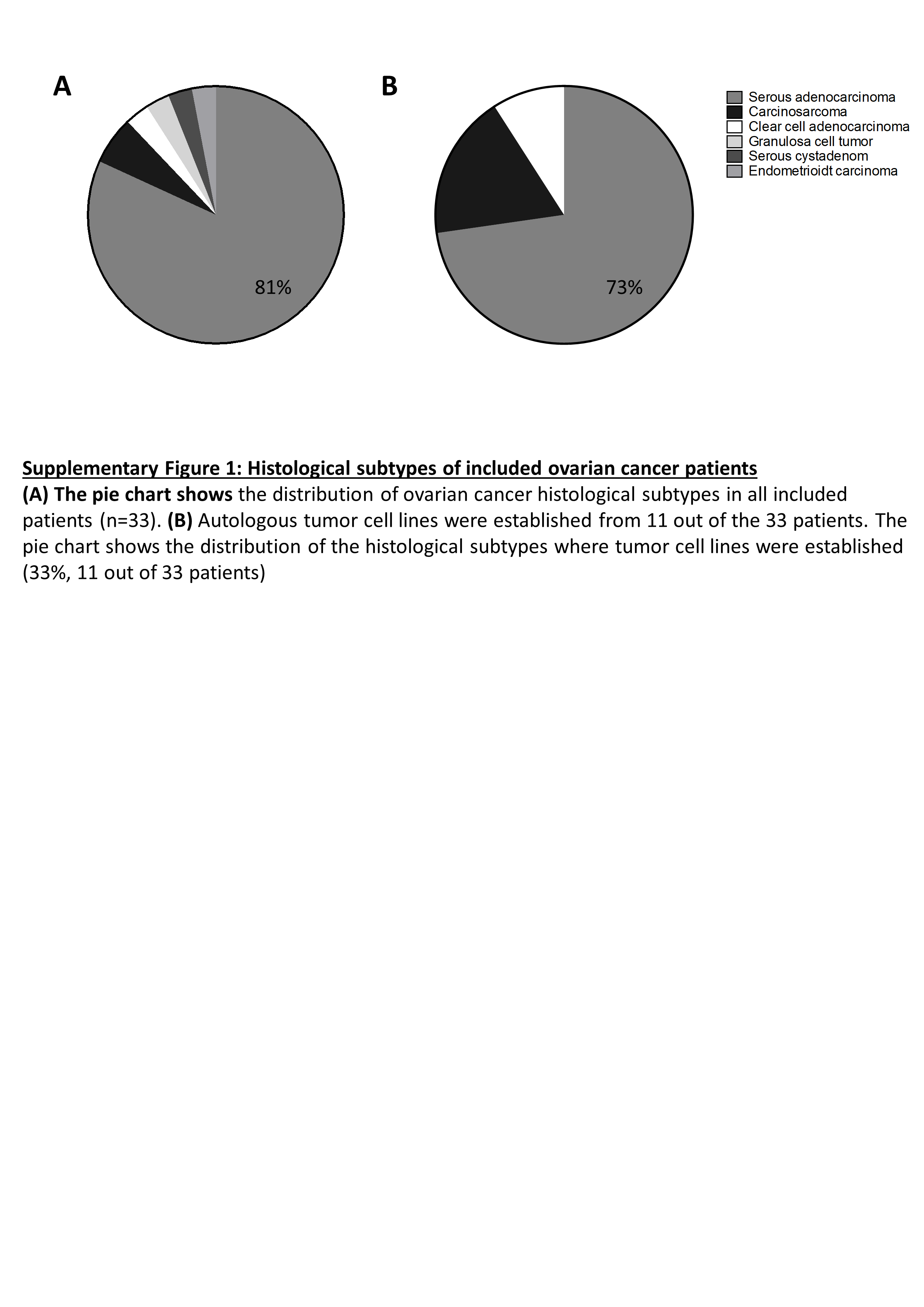

Supplement: Supplementary file 3 — Supplementary Figure 1 [file 41416_2019_384_MOESM3_ESM.tif]

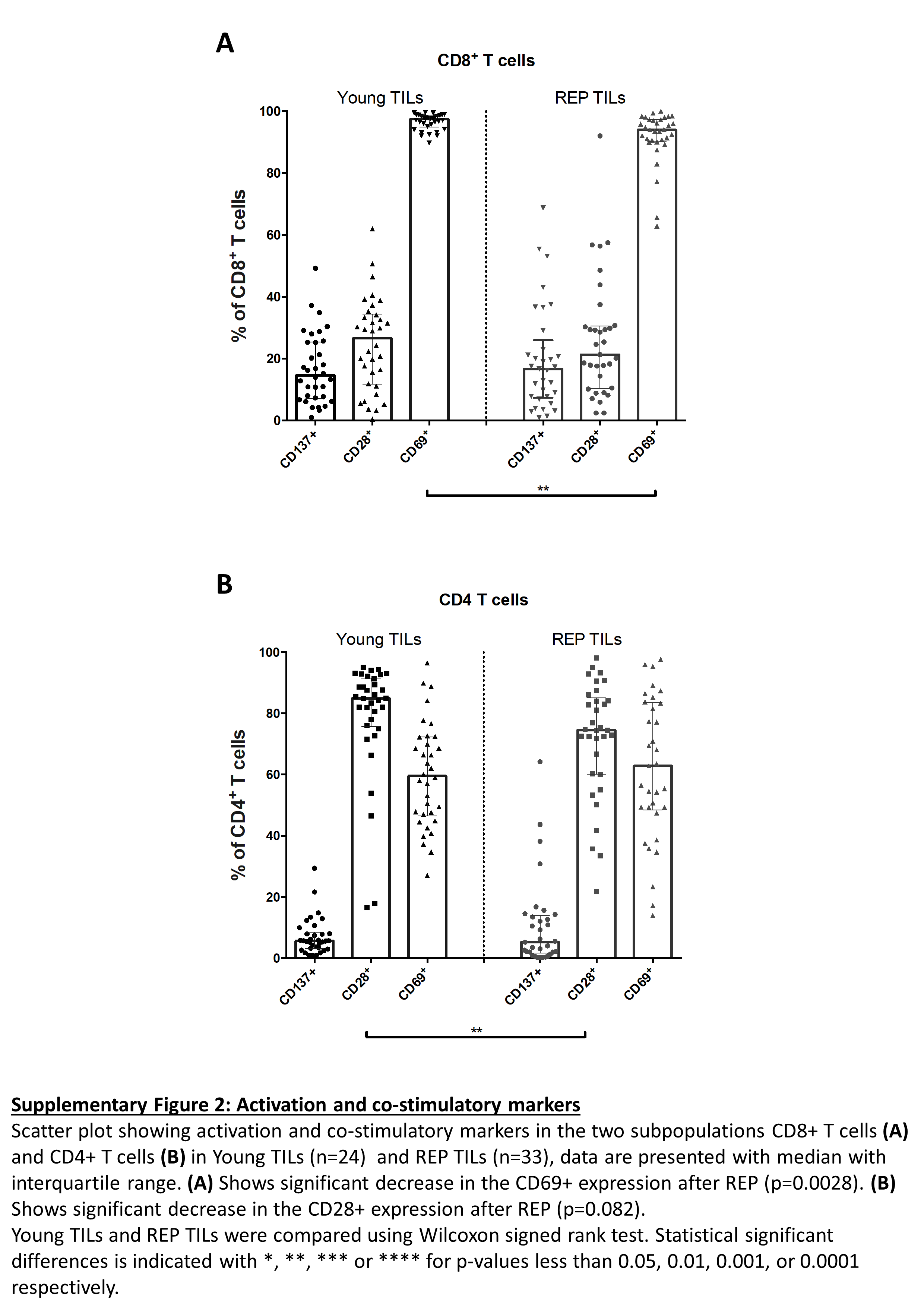

Supplement: Supplementary file 4 — Supplementary Figure 2 [file 41416_2019_384_MOESM4_ESM.tif]

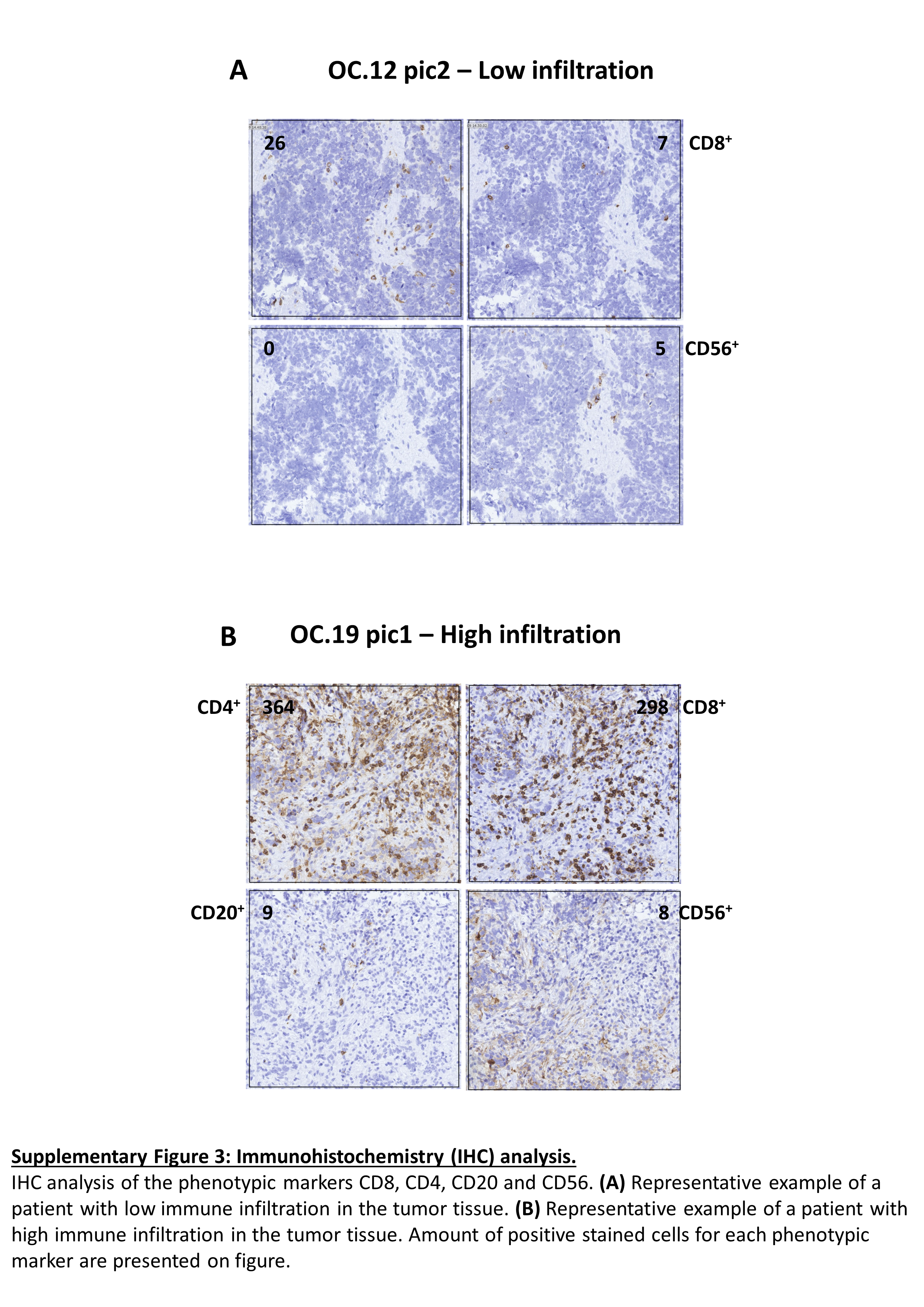

Supplement: Supplementary file 5 — Supplementary Figure 3 [file 41416_2019_384_MOESM5_ESM.tif]

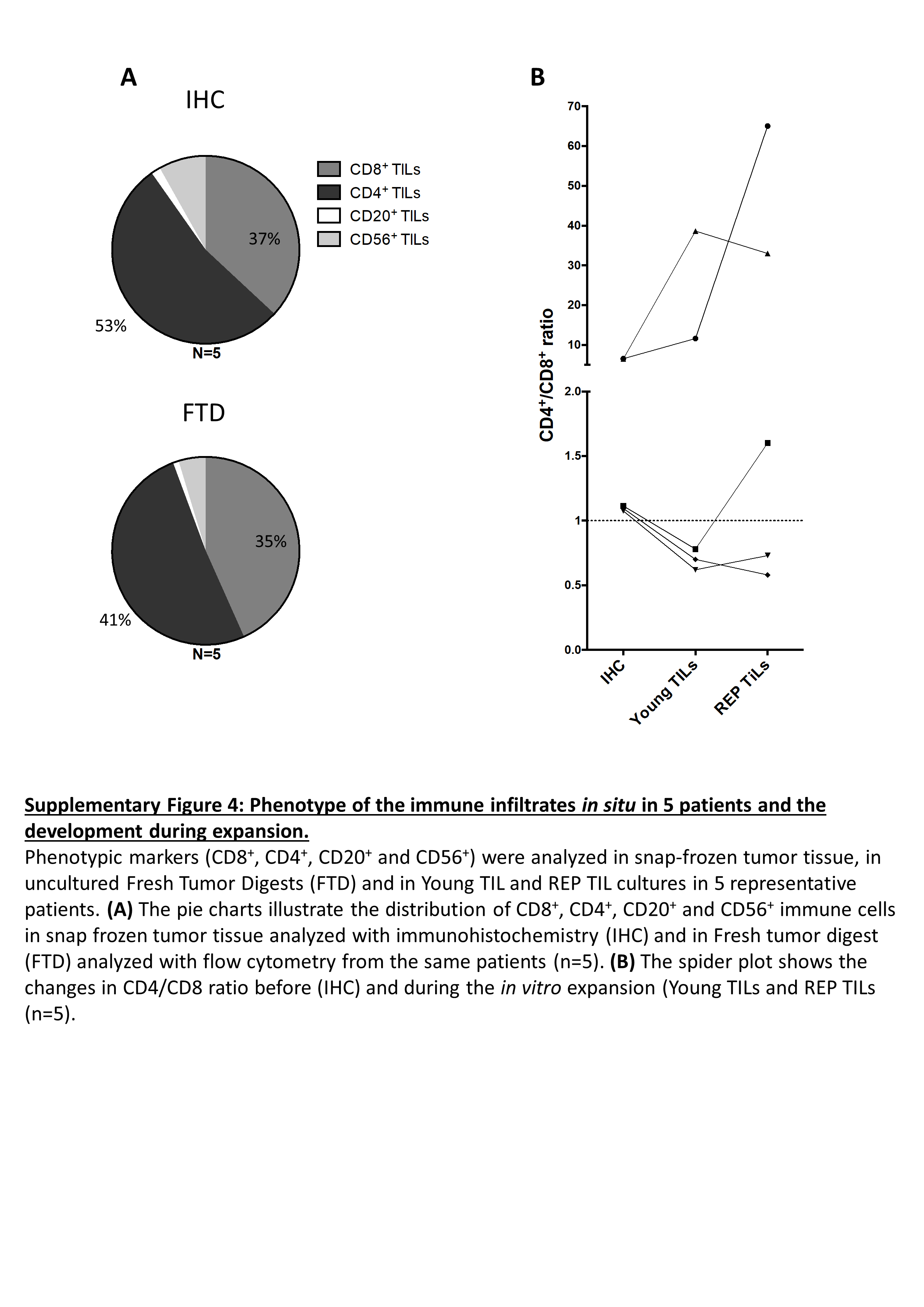

Supplement: Supplementary file 6 — Supplementary Figure 4 [file 41416_2019_384_MOESM6_ESM.tif]

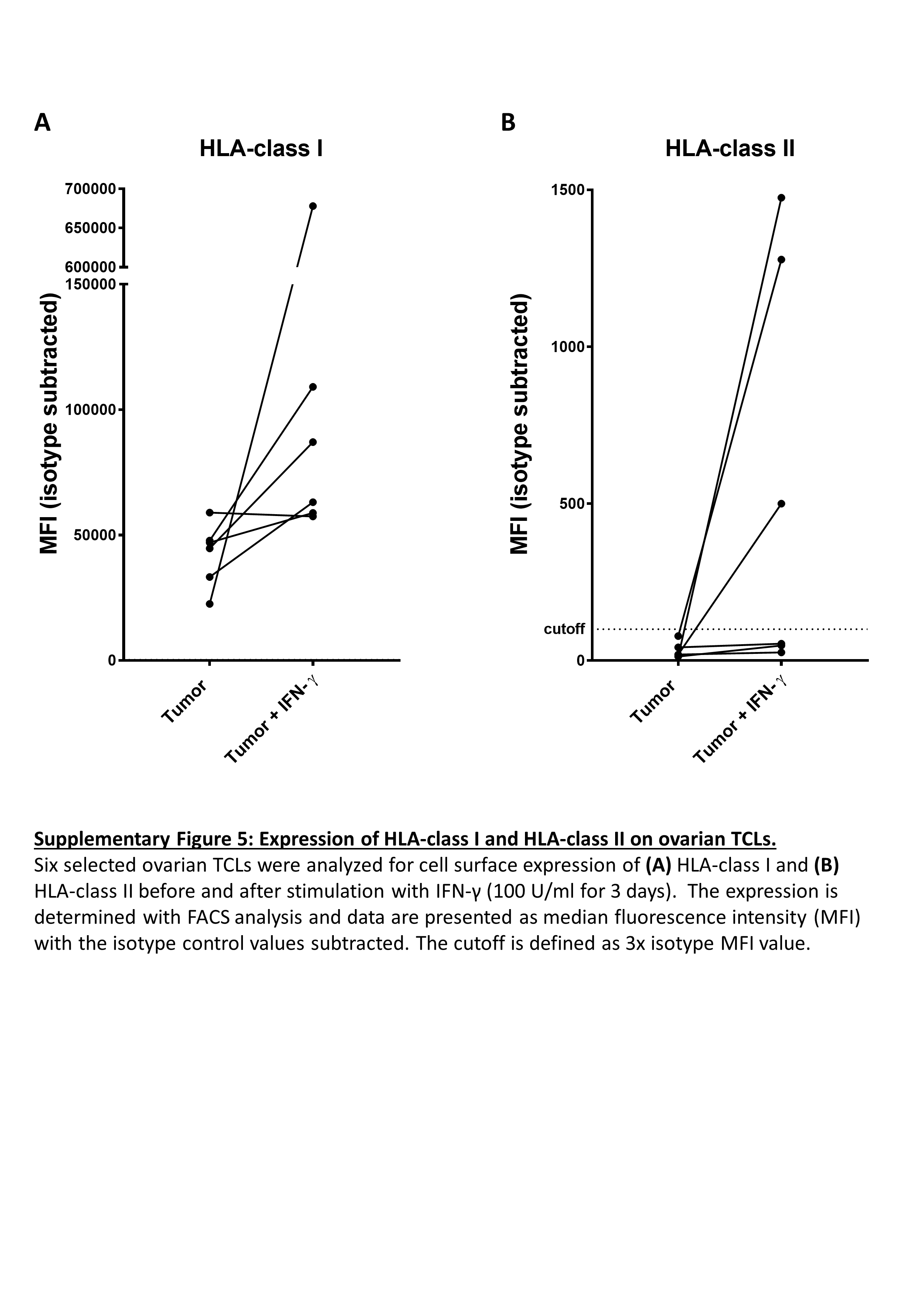

Supplement: Supplementary file 7 — Supplementary Figure 5 [file 41416_2019_384_MOESM7_ESM.tif]

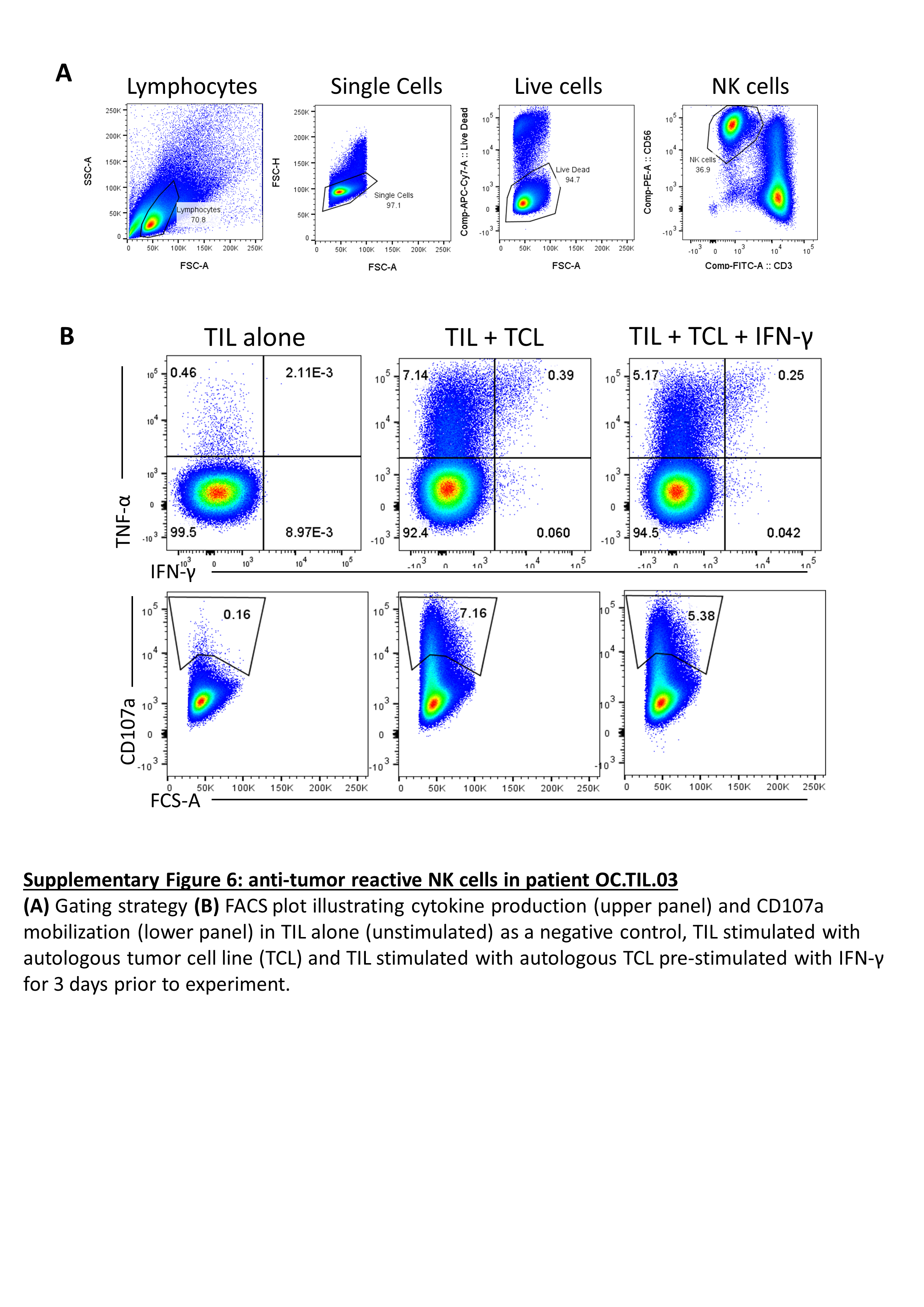

Supplement: Supplementary file 8 — Supplementary Figure 6 [file 41416_2019_384_MOESM8_ESM.tif]

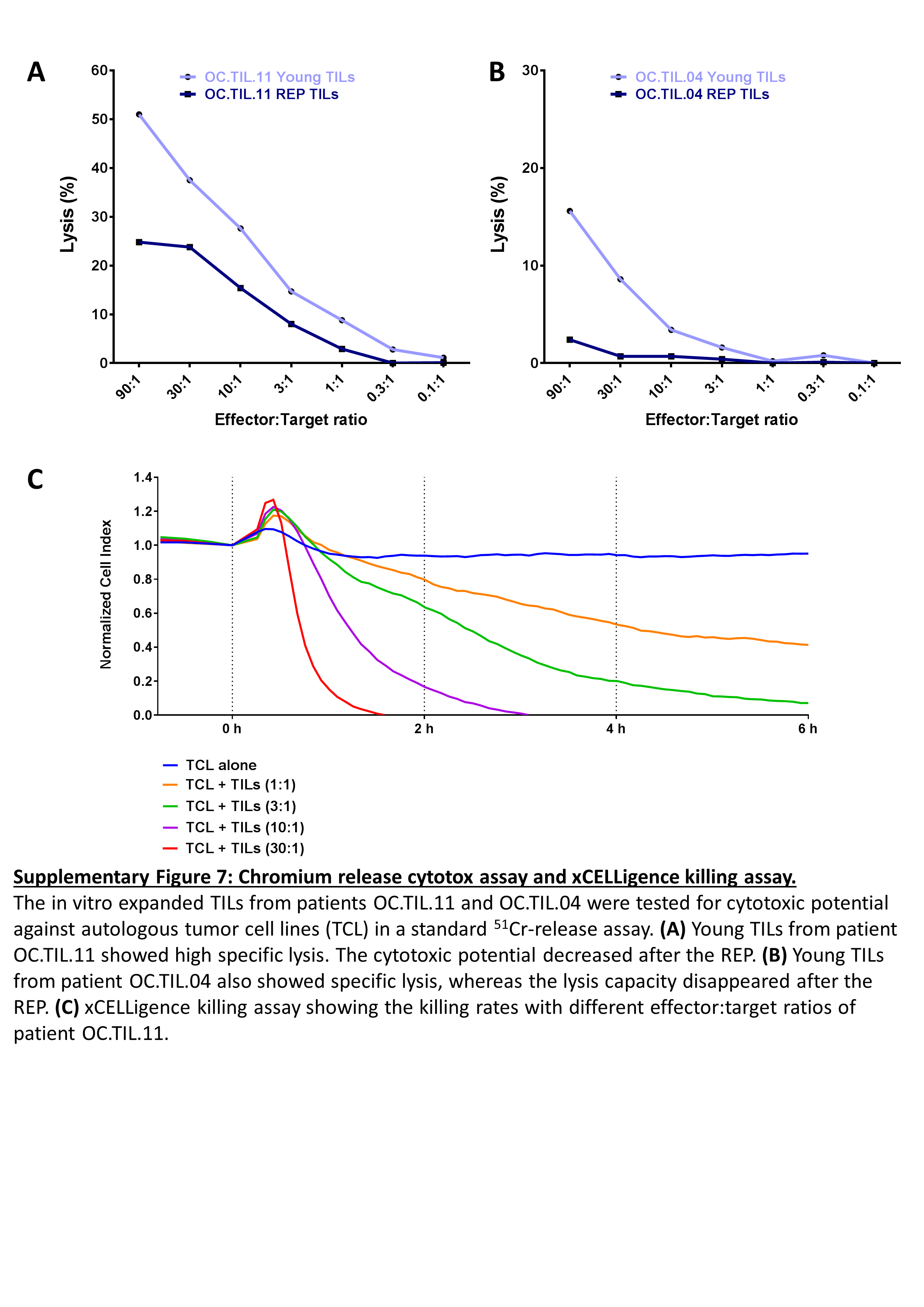

Supplement: Supplementary file 9 — Supplementary Figure 7 [file 41416_2019_384_MOESM9_ESM.tif]

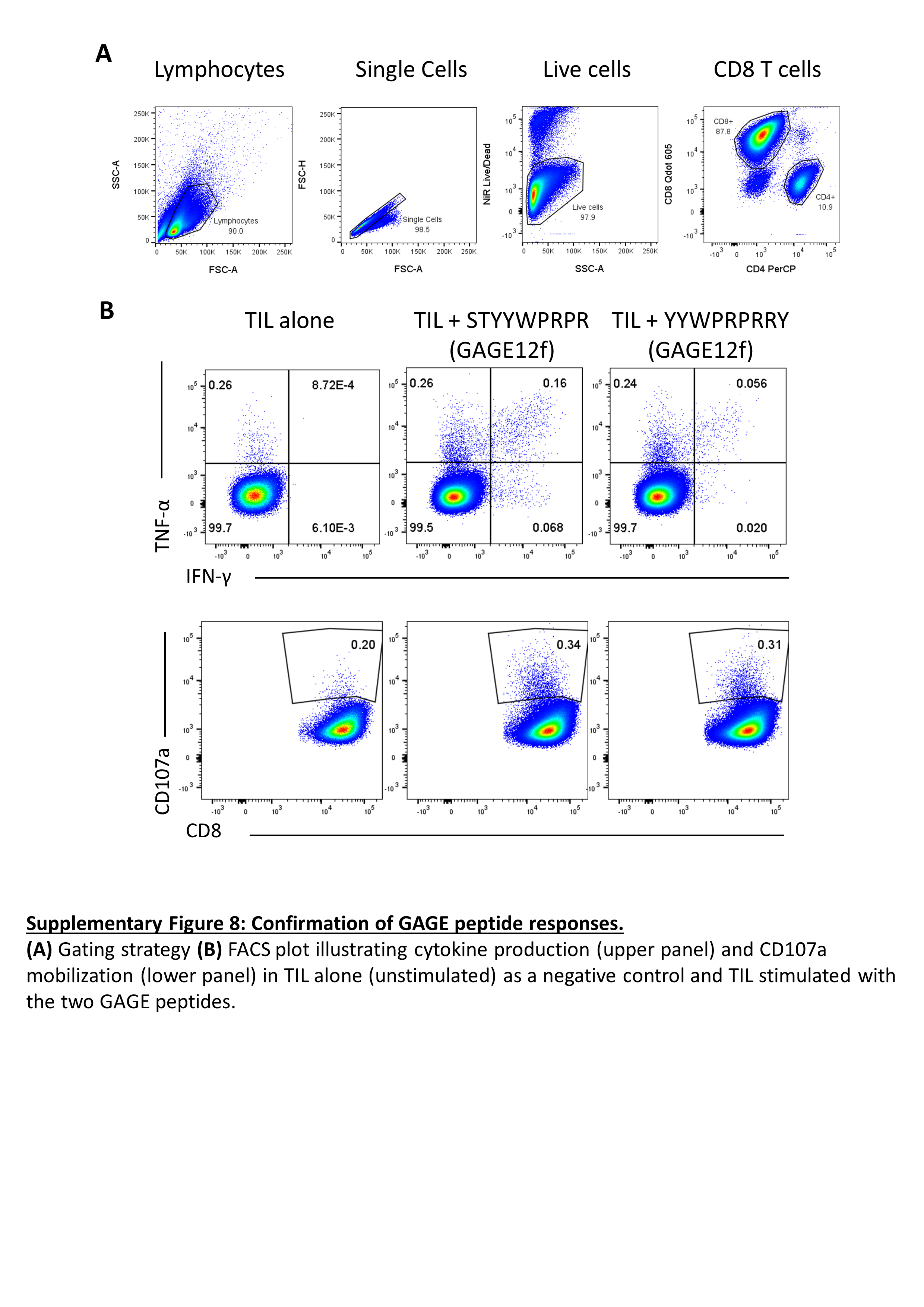

Supplement: Supplementary file 10 — Supplementary Figure 8 [file 41416_2019_384_MOESM10_ESM.tif]
