# Supplementary material for: Tumour-reactive T cell subsets in the microenvironment of ovarian cancer
Source: Br J Cancer. 2019 Feb 5;120(4):424–34. doi: 10.1038/s41416-019-0384-y (PMC6461863; doi:10.1038/s41416-019-0384-y)
Supplement: Supplementary file 2 — Supplementary Tables [file 41416_2019_384_MOESM2_ESM.docx]

| Patient ID. | Age | Histology | Autologous tumor cell line (X) | Ascites (X) | Age of Young TIL (days) | Young TIL CD4/CD8 | REP’d TIL CD4/CD8 | Fold expansion | Chemotherapy naïve / Chemotherapy treated before resection | Stage (FIGO) | Grading | Survival (months from OP) |
| --- | --- | --- | --- | --- | --- | --- | --- | --- | --- | --- | --- | --- |
| OC.TIL.01 | 63 | Ovarian serous adenocarcinoma |  | X | 24 | 5.37 | 4.25 | 567 | Naïve | IV | High grade | 29+ |
| OC.TIL.03 | 75 | Ovarian serous adenocarcinoma | X | X | 27 | 2.64 | 1.79 | 822 | Naïve | III | High grade | 29+ |
| OC.TIL.04 | 50 | Ovarian clear cell adenocarcinoma | X |  | 29 | 2.07 | 65.2 | 935 | Naïve | IIIb | High grade | 29+ |
| OC.TIL.05 | 70 | Endometrioidt cacinoma |  |  | 16 | 2.56 | 3.08 | 1926 | Naïve | IIIc | High grade | 27+ |
| OC.TIL.06 | 64 | Ovarian serous adenocarcinoma |  |  | 17 | 6.17 | 3.42 | 636 | Naïve | IIIc | High grade | 27+ |
| OC.TIL.07 | 44 | Ovarian serous adenocarcinoma | X | X | 21 | 3.41 | 2.32 | 1250 | Naïve | IIIb | High grade | 27+ |
| OC.TIL.08 | 56 | Ovarian serous adenocarcinoma |  |  | 51 | 3.20 | 8.09 | 588 | Naïve | IV | Low grade | 26+ |
| OC.TIL.09 | 80 | Ovarian serous adenocarcinoma | X | X | 36 | 2.55 | 1.86 | 440 | Naïve | IIIa | Low grade | 26+ |
| OC.TIL.10 | 52 | Ovarian serous cystadenom, borderline |  | X | 34 | 0.00 | 0.03 | 1416 | Naïve | II | Not graded | 24+ |
| OC.TIL.11 | 67 | Carcinosarcoma | X | X | 21 | 2.32 | 0.74 | 2570 | Naïve | IIb | High grade | 24+ |
| OC.TIL.12 | 59 | Carcinosarcoma | X | X | 42 | 11.62 | 65.07 | 1014 | Naïve | IIIc | Not graded | 24+ |
| OC.TIL.13 | 57 | Ovarian Granulosa cell tumor |  | X | 43 | 3.12 | 40.13 | 500 | Naïve | Ia | Not graded | 23+ |
| OC.TIL.14 | 67 | Ovarian serous adenocarcinoma |  |  | 15 | 7.55 | 6.38 | 1074 | Naïve | IIIb | High grade | 23+ |
| OC.TIL.15 | 44 | Ovarian serous adenocarcinoma |  |  | 32 | 5.40 | 10.99 | 3724 | Naïve | IIIc | High grade | 15 |
| OC.TIL.16 | 49 | Ovarian serous adenocarcinoma |  | X | 20 | 2.62 | 8.66 | 3400 | Naïve | IIIb | High grade | 22+ |
| OC.TIL.17 | 54 | Ovarian serous adenocarcinoma | X | X | 59 | 2.79 | 18.70 | 3610 | Naïve | IIIc | Low grade | 3 |
| OC.TIL.19 | 71 | Ovarian serous adenocarcinoma |  |  | 29 | 0.70 | 0.58 | 2055 | 3 series neoadj. Carbo/Tax | IIIc | High grade | 17+ |
| OC.TIL.20 | 63 | Ovarian serous adenocarcinoma |  |  | 22 | 2.17 | 5.59 | 1905 | 3 series neoadj. Carbo/Tax | IV | High grade | 17+ |
| OC.TIL.21 | 45 | Ovarian serous adenocarcinoma | X |  | 17 | 2.23 | 1.03 | 1470 | 3 series Carbo/Pacli | IIIc | High grade | 17+ |
| OC.TIL.22 | 83 | Ovarian serous adenocarcinoma |  |  | 43 | 13.74 | 3.47 | 1027 | 3 series Carbo | IV | High grade | 17+ |
| OC.TIL.23 | 56 | Ovarian serous adenocarcinoma |  |  | 36 | 1.40 | 2.32 | 1635 | 3 series neoadj. Carbo/Tax) | IV | High grade | 17+ |
| OC.TIL.24 | 66 | Ovarian serous adenocarcinoma | X |  | 35 | 9.13 | 21.05 | 793 | 3 series Carbo/Pacli | IIIc | High grade | Dead |
| OC.TIL.25 | 77 | Ovarian serous adenocarcinoma |  |  | 29 | 7.32 | 107 | 4069 | 6 series Carbo/Pacli in 2010 (5 years before OP) | III | High grade | 12 |
| OC.TIL.27 | 73 | Ovarian serous adenocarcinoma | X |  | 26 | 38.64 | 33.00 | 1519 | Naïve | IIIc | High grade | Dead |
| OC.TIL.28 | 86 | Ovarian serous adenocarcinoma |  |  | 23 | 0.62 | 0.73 | 5544 | 3 series Carbo | IIIc | Not graded | 14+ |
| OC.TIL.30 | 68 | Ovarian or peritoneal serous adenocarcinoma |  |  | 28 | 10.69 | 9.66 | 3481 | 3 series Carbo/Tax + 1 series Doxo/Carbo | IIIc | High grade | 13+ |
| OC.TIL.31 | 62 | Ovarian serous adenocarcinoma |  |  | 19 | 2.06 | 3.22 | 2650 | 3 series Carbo | IV | High grade | 13+ |
| OC.TIL.32 | 72 | Ovarian serous adenocarcinoma |  |  | 18 | 1.46 | 1.16 | 3387 | Naïve | IIIc | Not graded | 13+ |
| OC.TIL.33 | 71 | Ovarian serous adenocarcinoma | X |  | 19 | 9.62 | 13.32 | 2250 | 3 series Carbo/Tax | IVa | High grade | Dead |
| OC.TIL.34 | 59 | Ovarian serous adenocarcinoma |  |  | 42 | 0.78 | 1.60 | 1685 | 3 series neoadj. Carbo/Tax) | IV | High grade | 12+ |
| OC.TIL.35 | 64 | Ovarian serous adenocarcinoma | X | X | 28 | 1.96 | 2.51 | 2800 | Naïve | IIIc | High grade | 12+ |
| OC.TIL.36 | 64 | Ovarian serous adenocarcinoma |  |  | 28 | 2.44 | 5.34 | 2496 | Naïve | IIIc | Low grade | 12+ |
| OC.TIL.37 | 75 | Ovarian serous adenocarcinoma |  | X | 31 | 6.37 | 18.35 | 1602 | 3 series neoadj. 1 serie Carbo/Tax, 2 series Carbo | IIIc | High grade | 11+ |
| OC.TIL.04 2nd | 52 | Ovarian clear cell adenocarcinoma |  |  | 21 | 0.21 | 0.07 | 4186 | 6 series Carbo/Tax | IIIb | High grade | 13+ |

**Supplementary Tabel 1: Clinical Characteristics**

| HLA peptide | Source protein | MaxQuant Score | HLA restriction (>2% rank) | Predicted Affinity (nM) | Immunogenic |
| --- | --- | --- | --- | --- | --- |
| ALDEKLLNI | CPSF1 | 84.227 | A02:01 | 19.3 | - |
| ALHKPPLHH | CPSF1 | 107.9 | A03:01 | 438.3 | - |
| EETVSGLKGY | CPSF1 | 87.754 | B44:02 | 130.5 | **-** |
| QELLIYEA | CPSF1 | 79.713 | NaN | NaN | **-** |
| RLGNSLLLK | CPSF1 | 94.407 | A03:01 | 9.9 | **-** |
| SEETVSGLKGY | CPSF1 | 76.221 | B44:02 | 238.3 | **-** |
| SLAEEHEGL | CPSF1 | 76.221 | A02:01 | 41 | **-** |
| HRWAQPDTA | KIF20A | 153.73 | B39:06 | 295.4 | **-** |
| KLQGQVSAK | KIF20A | 143.04 | A03:01 | 25.2 | **-** |
| KMLEPPPSAK | KIF20A | 101.38 | A03:01 | 12.3 | **-** |
| RVFQGFFTGR | KIF20A | 191.45 | A03:01 | 64.5 | - |
| SQLDETSHRW | KIF20A | 77.662 | B44:02 | 631.7 | - |
| STYYWPRPR | GAGE12F;GAGE6;GAGE5;GAGE4;GAGE12I;GAGE12G;GAGE7;GAGE3;GAGE1;GAGE12H;GAGE12B;GAGE12J | 133.6 | A03:01 | 57.8 | + |
| TQRQDPAAA | GAGE12F;GAGE6;GAGE5;GAGE4;GAGE12I;GAGE12G;GAGE7;GAGE3;GAGE1;GAGE12J;GAGE2D;GAGE2A;GAGE2E;GAGE2B;GAGE10 | 74.301 | NaN | NaN | - |
| YYWPRPRRY | GAGE12F;GAGE6;GAGE5;GAGE4;GAGE12I;GAGE12G;GAGE7;GAGE3;GAGE1;GAGE12H;GAGE12B | 78.616 | C07:02 | 12.7 | + |
| KLPGDKLGR | BRDT | 84.227 | NaN | NaN | **-** |
| DHWDSKNVSC | EZH2 | 101.43 | B39:06 | 1299.1 | **-** |
| SVNPNCYAK | EZH2;EZH1 | 100.19 | A03:01 | 345. 5 | **-** |
| ISLSNNTGKN | FAM46D | 96.492 | NaN | NaN | **-** |
| KEVDPAGHSY | MAGEA9;MAGEA8 | 203.19 | B44:02 | 67.9 | **-** |
| LLKSVGAQK | MDM2 | 83.168 | A03:01 | 69.7 | **-** |
| SETLKHLVL | NXF5;NXF2 | 84.244 | B44:02 | 1316.9 | **-** |
| KLLDAGGDLRL | TEX14 | 59.496 | A02:01 | 128.8 | **-** |

**Supplementary Table 2**. Selected MS-identified tumor-associated HLA peptides were tested for their immunogenicity in vitro. Predicted binding affinities were computed by NetMHC pan 4.0.
